# Complete metabolic response in stage IV Merkel cell carcinoma after combined avelumab and radiotherapy: a case report suggestive of an abscopal effect

**DOI:** 10.3389/fonc.2026.1867799

**Published:** 2026-08-06

**Authors:** Jordi Guzman-Casta, Samuel Alejandro Lozano-Valdez, Frida Sophia Trejo-Mendoza, Mariana Cecilia Macías-Flores

**Affiliations:** 1 Oncology Institute of Querétaro, Hospital Angeles Centro Sur, Querétaro, Mexico; 2 School of Medicine, Anáhuac University Querétaro, Querétaro, Mexico; 3 Radio-Oncology Department, Hospital Angeles Centro Sur, Querétaro, Mexico

**Keywords:** abscopal effect, avelumab, case report, immunotherapy, Merkel cell carcinoma, radiotherapy

## Abstract

Merkel cell carcinoma (MCC) is an aggressive neuroendocrine skin cancer with limited therapeutic options once it reaches metastatic stages. While immune checkpoint inhibitors like avelumab have improved outcomes, achieving a complete and durable response in stage IV disease remains challenging. We report the case of a 75-year-old male diagnosed with stage IV MCC, negative for Merkel cell polyomavirus (MCPyV) and with 1% PD-L1 tumor cell expression, presenting with a primary tumor in the right thigh, a regional inguinal nodal conglomerate, and a distant mediastinal lymph node metastasis. Initial treatment with first-line avelumab resulted in a discordant response, with a near-complete metabolic response of the mediastinal lesion but persistence of the primary tumor. Localized radiotherapy (RT) — 66 Gy in 33 fractions delivered by intensity-modulated radiotherapy (IMRT), encompassing the primary tumor, the inguinal nodal conglomerate, and at-risk regional nodal basins — was subsequently added while avelumab was continued. Following completion of RT and the fourteenth cycle of avelumab, a complete metabolic and morphological response was documented by FDG PET-CT across all disease sites, including the non-irradiated mediastinal node, consistent with an abscopal effect. Treatment was reasonably well tolerated, with grade 3 radiodermatitis and grade 1 radiation cystitis as the only reported adverse events, and no immune-related toxicity. The patient has since maintained a durable remission under surveillance with maintenance RT. This case suggests a potent synergistic interaction between avelumab and RT, potentially mediated by an abscopal effect at the non-irradiated distant site. The strategic combination of these therapies may overcome initial resistance to immunotherapy in advanced MCC, highlighting the need for further clinical investigation into radio-immunotherapy protocols for this malignancy.

## 1 Introduction

Merkel cell carcinoma (MCC) is a highly aggressive neuroendocrine skin malignancy characterized by rapid growth, early metastatic spread, and poor outcomes in advanced disease, its incidence is 0.7 cases per 100,000 persons in the USA. Its pathogenesis is primarily associated with clonal integration of the Merkel cell polyomavirus (MCPyV) into the host genome and chronic exposure to UV radiation (1). Before the introduction of immune checkpoint inhibitors (ICI), systemic treatment relied primarily on platinum-based regimens. Although initial responses were frequently observed, these were typically short-lived and rarely translated into durable disease control (2, 3).

The introduction of ICI such as avelumab, has changed the therapeutic landscape of advanced MCC. Avelumab is an IgG1 monoclonal antibody that targets programmed death-ligand 1 (PD-L1), restoring antitumor immune responses by blocking the PD-1/PD-L1 interaction between tumor cells and T lymphocytes. The phase II JAVELIN Merkel 200 trial reported response rates of 30% in previously treated patients and close to 40% when used as the first-line treatment. These responses were often durable, establishing avelumab as a standard systemic therapy for patients with advanced or metastatic MCC (4).

Radiotherapy (RT) is an important component in the management of MCC due to the radiosensitivity of this tumor. Beyond its local cytotoxic effects, RT may also enhance systemic antitumor immune responses through the release of tumor-associated antigens and activation of inflammatory pathways within the tumor microenvironment. These mechanisms have been proposed to underlie the Abscopal effect, a phenomenon in which localized irradiation results in regression of distant, non-irradiated tumor lesions (5).

We present the case of a patient with metastatic MCC treated with avelumab and RT who achieved a complete metabolic response documented by FDG PET−CT, suggesting an abscopal effect.

## 2 Case presentation

A 75-year-old Hispanic male with a history of systemic arterial hypertension under pharmacological treatment presented for evaluation of a 5-month history of a progressively enlarging mass located in the right thigh. Initial ultrasonography (week 0) demonstrated a highly vascularized soft-tissue lesion suspicious for malignancy. A core needle biopsy was subsequently performed (week 1), and histopathological examination revealed findings consistent with MCC. Molecular characterization showed MCPyV negativity by PCR, PD-L1 expression of 1% in tumor cells, and tumor-infiltrating lymphocytes were not evaluated.

**Figure 1:**
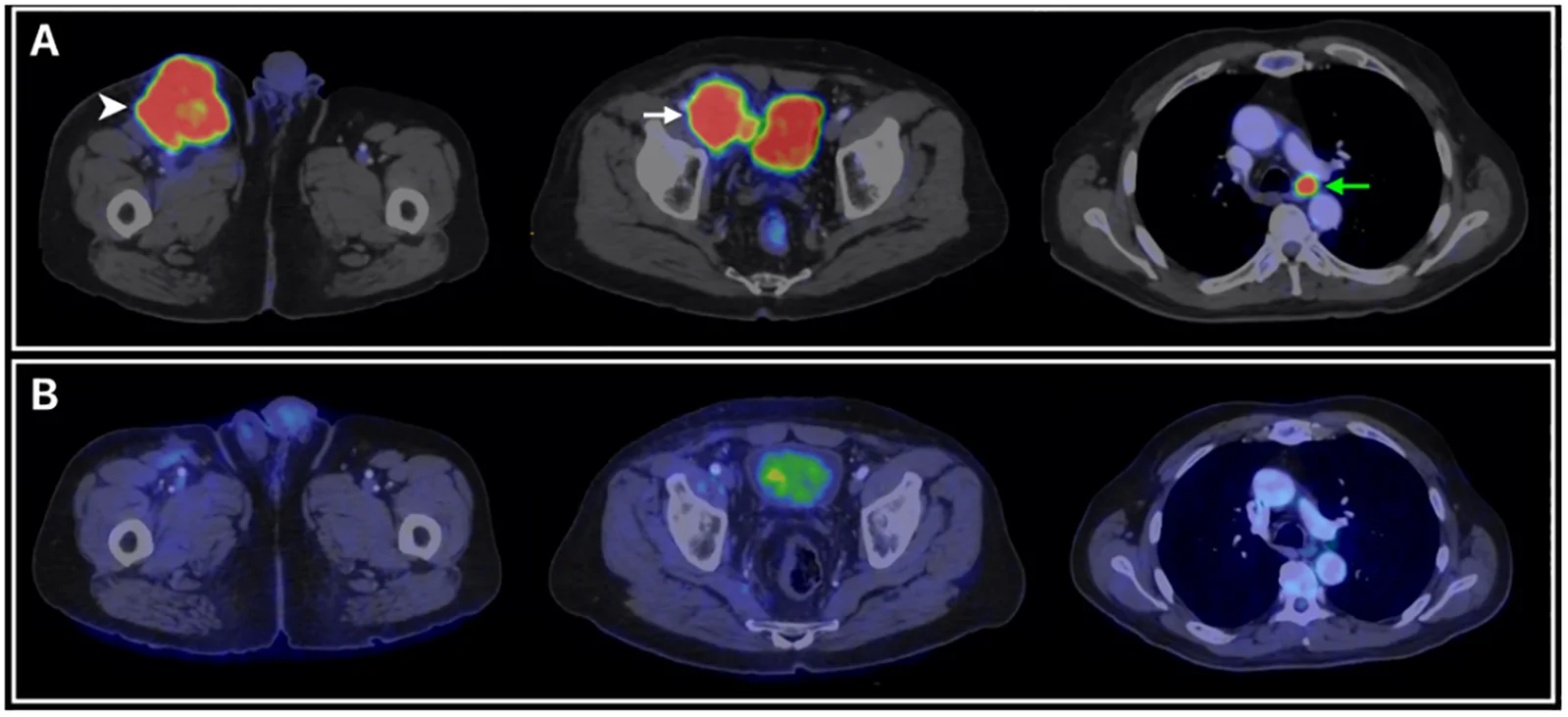
FDG PET-CT at baseline tumor activity and complete response to treatment. **(A)** Hypermetabolic solid mass in the right thigh (arrowhead), hypermetabolic lymph nodes conglomerate along the right external iliac chain (white arrow), hypermetabolic paratracheal lymph node (green arrow). **(B)** Complete metabolic and morphologic resolution of all lesions.

A baseline FDG PET-CT was obtained (week 2), demonstrating a hypermetabolic solid mass in the subcutaneous tissue of the right thigh measuring 97 × 75 × 72 mm with an SUVmax of 15.2, without evidence of infiltration of the anterior muscular compartment of the limb. Additionally, multiple hypermetabolic lymph nodes were identified, forming a conglomerate along the right external iliac chain measuring 58 × 56 × 52 mm with an SUVmax of 15.7. A hypermetabolic mediastinal lymph node was also observed in the inferior paratracheal region, measuring 21 × 16 mm with an SUVmax of 13.5, suggestive of distant metastatic involvement (**Figure 1A**).

Based on these findings, the patient was diagnosed with stage IV MCC due to mediastinal metastatic disease. First-line systemic therapy with avelumab 800 mg administered every two weeks was initiated at week 3. At week 7, a follow-up FDG PET-CT demonstrated a near-complete partial metabolic response according to iRECIST criteria at the mediastinal site; however, no significant metabolic response was observed in the primary tumor or the inguinal nodal conglomerate. Given this discordant response, systemic treatment with avelumab was continued, and RT was added to the primary tumor and regional nodal basins.

RT was delivered using intensity-modulated radiotherapy (IMRT) with a linear accelerator (EDGE), to a total dose of 66 Gy in 33 fractions (2 Gy per fraction). The treatment volume encompassed the primary/residual thigh tumor, the right inguinal nodal conglomerate, and at-risk regional nodal basins, including the left inguinal, internal obturator, and external obturator chains. By week 31, the patient had completed the 33 planned RT fractions and reached the fourteenth cycle of avelumab. At this point, RT was transitioned to maintenance sessions administered every 28 days — a decision made jointly by the multidisciplinary team and the patient; while no direct evidence supports this specific maintenance schedule, it was considered biologically plausible in the context of sustaining an abscopal-mediated immune response.

**Figure 2:**
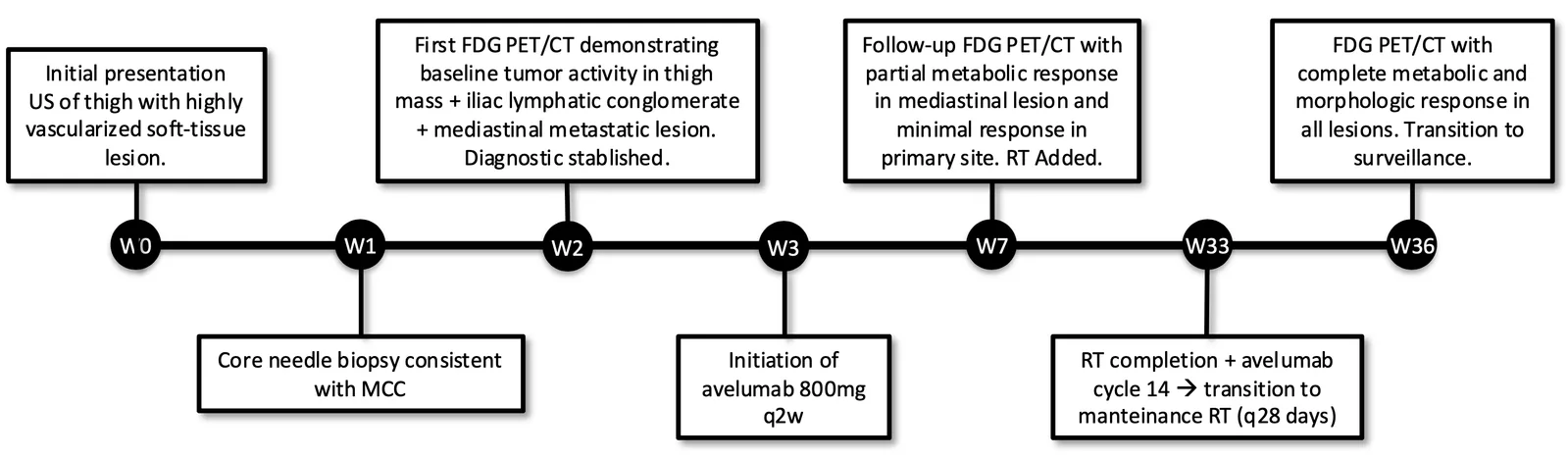
Clinical timeline of disease course and treatment. US, Ultrasonography; MCC, Merkel cell carcinoma; q2w, every two weeks; RT, Radiotherapy.

At week 36 (33 weeks after avelumab initiation), a follow-up FDG PET-CT demonstrated complete metabolic resolution of the right thigh mass, complete resolution of the right inguinal nodal conglomerate, and complete metabolic resolution of the mediastinal lymph node, which decreased to 11 × 11 mm on CT with no residual hypermetabolic activity. No evidence of residual metabolically active disease was observed at any site, consistent with a complete metabolic and morphological response (**Figure 1B**). Post-treatment changes in the right thigh were noted on imaging and initially described as suggestive of surgical changes; however, no surgical resection was performed at any point during management, and these findings were attributed to radiotherapy-induced fibrotic and inflammatory changes. Incidental calcified pulmonary granulomas, unrelated to MCC, were also noted. The patient was subsequently transitioned to surveillance with continued maintenance RT. **Figure 2** outlines the timeline of the patient's clinical progression and therapeutic management.

Regarding treatment tolerability, serial complete blood counts showed normal lymphocyte counts throughout treatment. Inflammatory markers obtained during the RT course were elevated, with C-reactive protein of 17.2 mg/L and erythrocyte sedimentation rate of 55 mm/h. No immune-related adverse events or clinical signs of systemic immune activation (e.g., fever) were observed. The only treatment-related toxicities were grade 3 radiodermatitis, controlled with topical corticosteroids at the primary tumor site, and grade 1 radiation cystitis, both graded according to CTCAE v5.0.

## 3 Discussion

This report describes a complete metabolic response in a patient with stage IV MCC following combined treatment with avelumab and RT. Although the primary tumor and the regional inguinal nodal conglomerate were included within the RT treatment volume, the mediastinal lymph node — a distant site that did not receive irradiation — also achieved complete metabolic resolution. This finding is consistent with a true abscopal effect, in which localized RT contributed to a systemic antitumor immune response capable of inducing regression at a non-irradiated distant site. Although ICI have significantly improved the prognosis of advanced MCC (4), achieving complete and durable responses in metastatic disease through combined strategies remains infrequent and poorly documented.

In this case, the patient initially presented a discordant response, characterized by a near-complete reduction in metabolic activity in the mediastinal lesion, in contrast to the persistence of active disease in the primary tumor and regional nodal conglomerate. Following the administration of targeted RT to the primary and regional sites, a complete metabolic resolution was observed across all disease sites, including the non-irradiated mediastinal node. While a direct causal relationship cannot be definitively established, the temporal sequence of these findings and the regression of the non-irradiated lesion suggest a potential synergistic effect between immunotherapy and RT.

From a biological perspective, RT can modulate the tumor microenvironment by inducing immunogenic cell death, releasing tumor neoantigens, and activating inflammatory signaling pathways that promote antigen presentation (5). In this context, PD-L1 blockade with avelumab may enhance the expansion and activity of previously activated cytotoxic T lymphocytes, facilitating a systemic immune response capable of impacting non-irradiated tumor sites such as the mediastinal node observed in this case. This mechanistic rationale — namely, that fractionated RT can upregulate PD-L1 expression and sustain local immune activation (5) — also informed the decision to continue RT in a maintenance schedule following the completion of the primary treatment course; however, no prospective evidence currently validates this specific regimen, and its use in this case reflects an individualized, biologically-motivated clinical decision rather than an established protocol.

To our knowledge, the specific clinical sequence of an initial discordant response followed by a complete metabolic remission mediated by the abscopal effect has not been previously reported in advanced MCC. Literature regarding this synergy is exceptionally sparse and differs meaningfully from our case. Xu et al. (6) reported abscopal responses using short-course RT administered as salvage therapy after patients had already progressed on PD-1 blockade; Torchio et al. (8) described an exceptional response to avelumab monotherapy, without RT, following failure of electrochemotherapy; and Principe et al. (7) reported complete regression with combined radio-immunotherapy but without a discordant-response pattern or quantitative iRECIST-based documentation. Our case is distinct in describing RT added proactively during first-line avelumab in the setting of a clearly documented discordant response, using conventionally fractionated RT, with quantitative SUVmax and iRECIST-based documentation at all three disease sites. This rare but reproducible phenomenon underscores the clinical importance of documenting such interactions to refine therapeutic strategies for advanced, treatment-resistant MCC.

An important alternative explanation for the systemic response observed in this case is that it represents a delayed, intrinsic effect of avelumab monotherapy, independent of RT. In the JAVELIN Merkel 200 trial (9), although the median time to response to avelumab was 6.1 weeks, individual responses were reported as late as 36 weeks after treatment initiation, with approximately one-quarter of responses occurring beyond the 6-week landmark [new ref]. In our patient, the complete metabolic response was documented at week 36 of avelumab therapy, a time point that falls within the reported range of late avelumab-only responses; therefore, a delayed pharmacodynamic effect of avelumab cannot be excluded, and the temporal association between the addition of RT and the eventual complete response does not, by itself, establish causality. Nonetheless, the marked contrast between the near-absent response of the primary tumor and inguinal conglomerate after four weeks of avelumab monotherapy and their subsequent complete resolution following RT, together with the biological plausibility of RT-induced immune sensitization, favors a contributory role of RT in this case. Distinguishing between these possibilities would require either a treatment interruption — not clinically justifiable in this patient — or a larger comparative series, neither of which is feasible from a single case report.

This report has limitations inherent to its design, including the absence of a formal immunological biomarker panel beyond baseline PD-L1 expression and the fact that tumor-infiltrating lymphocytes and genomic/transcriptomic characterization (mutational burden, immune expression signatures) were not evaluated, the latter due to resource constraints. However, serial monitoring with FDG PET-CT provided an objective and quantifiable assessment of the tumor’s metabolic response, strengthening the validity of the clinical findings.

From a clinical standpoint, this case generates the hypothesis that the addition of localized RT in patients with a discordant response to first-line avelumab may convert an incomplete response into a complete, durable systemic response — a strategy that, if validated, could offer a practical approach for the subset of patients with MCC who achieve partial or discordant responses to ICI monotherapy.

Taken together, this case suggests a potential abscopal effect in the management of advanced MCC and highlights the need for further studies to define the role of these combined strategies in clinical practice.

## 4 Conclusion

In conclusion, this case demonstrates that the combination of avelumab and RT can induce a durable complete metabolic response in metastatic MCC, including regression of a non-irradiated distant lesion, suggesting an abscopal effect. While immunotherapy has transformed the prognosis of this malignancy, the strategic integration of RT in patients with discordant responses represents a promising therapeutic avenue. Prospective studies are warranted to validate the efficacy and safety of this combination and to identify biomarkers that may predict which patients will derive the greatest benefit from this radio-immunological approach.

## Data Availability

The original contributions presented in the study are included in the article/supplementary material. Further inquiries can be directed to the corresponding author.
